# Editorial: Fragmentation in Sleep and Mind: Linking Dissociative Symptoms, Sleep, and Memory

**DOI:** 10.3389/fpsyg.2017.02248

**Published:** 2017-12-22

**Authors:** Dalena van Heugten - van der Kloet, Sue Llewellyn

**Affiliations:** ^1^Faculty of Health and Life Sciences, Department of Psychology, Health & Professional Development, Oxford Brookes University, Oxford, United Kingdom; ^2^Nuffield Department of Clinical Neurosciences, The Sir William Dunn School of Pathology, Sleep and Circadian Neuroscience Institute, University of Oxford, Oxford, United Kingdom; ^3^Faculty of Humanities, University of Manchester, Manchester, United Kingdom

**Keywords:** dissociation, unusual sleep experiences, memory, de-differentiation, hyperassociative thinking

Dissociative symptoms are notorious for their enigmatic, disparate nature encompassing excessive daydreaming, memory problems, absentmindedness, and impairments and discontinuities in perceptions of the self, identity, and the environment. Recent studies (e.g., Koffel and Watson, 2009) have linked dissociative symptoms to vivid dreaming, nightmares, and objective sleep parameters (e.g., lengthening of REM sleep) for discussion, see Van der Kloet et al. (2013). Germane to this link between dissociative symptomology and sleep, is the idea that in dissociative individuals, the waking state as compared to REM sleep may be marked by an increase in “fluid” and hyperassociative thinking.

Against this background, we invited contributions in the following areas: (1) a progressive and enduring de-differentation of wake and dream states of consciousness eventually results in schizophrenia (Llewellyn, 2011); a lesser degree of de-differentiation may have implications for dissociative symptoms; (2) sleep disturbances are not only linked to dissociation but to memory fragmentation also, further fuelling both dissociation and other manifestations of psychopathology; (3) sleep, memory and psychopathologies have complex interlinkages.

Our summary below, relates the contributions to our topic to these areas. The contributing articles also give a comprehensive overview on current directions and challenges in these research fields.

First, Soffer-Dudek elucidates why heightened sleep experiences (vivid dreams, nightmares, hypnagogic hallucinations) might be particularly associated with psychopathology. She explains how various psychopathologies may represent de-differentiation of the waking and dreaming states through intrusions of wake-like consciousness into sleep and dream-like cognition into wake. With regard to de-differentiation, she discusses theories of transliminality (“thin boundaries between consciousness states”) and dissociation as potential mechanisms. Lucid dreaming is a hybrid state where wake-like cognition suffuses REM sleep/dreaming (Voss et al., 2009). Mota et al. compared the spontaneous occurrence of lucid dreaming in patients with psychotic symptoms (25 with schizophrenia and 20 with bipolar disorder) and 28 non-psychotic subjects. They found lucid dreaming was associated with psychosis- giving some support to the hypothesis of a link between state de-differentiation and psychopathology. In a methodological note, Ribeiro et al. having studied the prevalence of lucid dreaming using two questionnaires, show that the type of methodology used in lucid dreaming studies may have an effect upon their findings. Poerio et al. state that both research and theory points to dissociation being engendered by the intrusion of dream-like mentation into waking consciousness. To extend this idea they examine the role of sleep and daydreaming as potentiating states for subsequent dissociation in depersonalization/derealization disorder (DDD). They show the occurrence and content of daydreams may act as potentiating states for heightened, in the moment, dissociation. Sleep paralysis is a dissociated state where a heightened level of wake-like alertness coexists with muscle atonia. de Sá and Mota-Rolim take a broad interdisciplinary perspective on sleep paralysis through reviewing its occurrence in Brazilian folklore by exploring the “Pisadeira” with a sleep science approach and link this with the field of history and art.

There is now substantial evidence that active memory processing continues during sleep (see, for example, Rasch and Born, 2013) and dreaming may be involved (Llewellyn, 2013). De-differentiation of the wake, sleep and dream states would disrupt this memory processing fuelling dissociation and other psychopathologies. Horton presents an overview of discontinuity of consciousness in both wake and sleep, focusing on the processes of sleep-dependent memory consolidation and fragmentation. Nakagawa et al. focus specifically on working memory and the relation with sleep. With their article, they are the first to investigate differences between verbal working memory (VWM) and visuospatial working memory (VSWM) related to daytime nap duration, nap frequency, and dream content recall frequency (DCRF). They discuss sex-related differences in the effects of sleep habits on VMW and VSWM and ascribe this to differences in underlying neural correlates, and effectiveness of sleep habits in males and females. Rosales-Lagarde et al. related to emotional memory by designing an extended International Affective Picture System (IAPS) instrument to measure bizarreness and show some interesting age and gender disparities.

Finally, several authors took a developmental approach through focusing on sleep, memory and psychopathology in adolescents. Wang et al. show the relation between children and adolescents' mental health and sleep problems in a 10-year longitudinal trial, and find anxiety, depression, attention problems and aggressive behavior during childhood to be important predictors for later sleep problems. Importantly, sleep problems are also predictive of behavioral difficulties later in life. Nader et al. investigated changes in sleep in adolescents between 12 and 19 years old following procedural task training and found that contrary to earlier work, there were no changes in sleep spindle density. Interestingly, participants who successfully learned the task showed no changes in their sleep stage proportions, but participants who were not successful showed a decrease in the proportion of Stage 2 and increases in both SWS and REM sleep, which is in line with the two stage model of sleep and memory by Smith et al. (2004).

The self and the world during dreaming differ. The perceived world during waking depends on making sense of external sensory input, which engenders a strong sense of external reality in which the “self and its inner-world” exists. Self-organizing during waking and dreaming enables integration of the self and the world in both states. It is noteworthy, that within this research topic so many novel themes have emerged and new questions and speculations have been posed with regards to relations among unusual sleep experiences—specifically lucid dreaming—, dissociation, psychosis, and memory. It brings together a richness of research by combining fields that originally have worked in isolation from each other.

## Author contributions

Both authors contributed to writing up the topic, handling the reviews and writing up the editorial.

### Conflict of interest statement

The authors declare that the research was conducted in the absence of any commercial or financial relationships that could be construed as a potential conflict of interest.
